# Peripheral Neuropathy Expands the Neurological Phenotype in Glutaric Aciduria Type 1

**DOI:** 10.1002/jimd.70131

**Published:** 2026-01-04

**Authors:** Fabian Preisner, Sven F. Garbade, Inga Harting, Saskia B. Wortmann, Chris Mühlhausen, Sabine Heiland, Martin Bendszus, Stefan Kölker, Nikolas Boy

**Affiliations:** ^1^ Department of Neuroradiology Heidelberg University Hospital Heidelberg Germany; ^2^ Division of Pediatric Neurology and Metabolic Medicine, Department of Pediatrics I, Heidelberg University Hospital and Heidelberg University Medical Faculty of Heidelberg Heidelberg Germany; ^3^ University Children's Hospital, Paracelsus Medical University Salzburg Austria; ^4^ Department of Paediatrics and Adolescent Medicine University Medical Centre Göttingen, Georg‐August University Göttingen Germany; ^5^ Clinic for Child Neurology and Social Pediatrics, Children Centre Maulbronn Maulbronn Germany

**Keywords:** biochemical subtype, glutaric acidemia type 1, glutaric aciduria type 1, MR neurography, MRI, neurophysiology, polyneuropathy

## Abstract

Glutaric aciduria type 1 (GA1) is a neurometabolic disorder characterized by striatal injury in infancy and extrastriatal central nervous system abnormalities, the latter depending on the biochemical subtype. Whether the peripheral nervous system (PNS) is also affected has not been systematically studied. Therefore, we conducted a cross‐sectional study of 21 GA1 patients (15 high excretor [HE], 6 low excretor [LE]), identified either by newborn screening (NBS, *n* = 11) or targeted metabolic diagnostics (TMD, *n* = 10). All underwent clinical evaluation, cerebral MRI, neurophysiology, and MR‐neurography (MRN) of the sciatic nerve with magnetization transfer imaging and diffusion tensor imaging (DTI). Nerve magnetization transfer ratio (MTR) was analyzed across subgroups and against 21 age‐matched controls, while fractional anisotropy (FA) was assessed within the patient cohort. MRN revealed frequent abnormalities in GA1, particularly among HE patients, who showed lower MTR and FA values, indicating neuropathic changes. These alterations correlated with age, extrastriatal MRI abnormalities, and subependymal nodules, but not with striatal lesions or movement disorder. Clinical neuropathic symptoms were rare (4/15 HE patients) yet consistently associated with abnormal MRN. In HE patients exclusively, neurophysiology demonstrated reduced compound motor action potentials, slowed nerve conduction, and prolonged tibial somatosensory evoked potential latencies. Within the HE subgroup, NBS‐identified patients showed higher MTR values than those identified by targeted metabolic diagnostics, suggesting less severe nerve involvement. These results expand the GA1 phenotype by demonstrating frequent, predominantly subclinical PNS involvement in HE patients, linked to chronic metabolic toxicity. They underscore the need for further research into long‐term complications and therapeutic strategies for HE individuals.

**Abbreviations**
3‐OH‐GA: 3‐hydroxyglutaric acid
AEC: acute encephalopathic crisis
CMAP: compound muscle action potential
DTI: diffusion tensor imaging
FA: fractional anisotropy
GA: glutaric acid
GA1: glutaric aciduria type 1
GCDH: glutaryl‐CoA dehydrogenase
HE: high excretor
LDA: linear discriminant analysis
LE: low excretor
MD: movement disorder
MRI: magnetic resonance imaging
MRN: magnetic resonance neurography
MTI: magnetization transfer imaging
MTR: magnetization transfer ratio
NBS: newborn screening
NCV: nerve conduction velocity
PNS: peripheral nervous system
SEP: somatosensory evoked potential
SNAP: sensory nerve action potential
TMD: targeted metabolic diagnostics

## 1 Introduction

Glutaric aciduria type 1 (GA1; OMIM #231670) is a rare neurometabolic disorder of lysine, hydroxylysine, and tryptophan metabolism caused by biallelic pathogenic variants in the *GCDH* gene, encoding the mitochondrial matrix protein glutaryl‐CoA dehydrogenase (GCDH, EC 1.3.8.6) [1, 2]. Decreased GCDH activity results in the accumulation of the neurotoxic metabolites glutaryl‐CoA, glutaric acid (GA), 3‐hydroxyglutaric acid (3‐OH‐GA), and non‐toxic glutarylcarnitine (C5DC), which is used for tandem mass spectrometry (MS/MS)‐based newborn screening (NBS) [3, 4, 5, 6, 7]. Two biochemical subtypes, that is, high and low excretors (HE, LE), have been defined depending on urinary GA concentrations and inversely correlated to residual enzyme activity [8].

Untreated individuals classically present with acute neurologic symptoms in early childhood. During a vulnerable period, most commonly between 3 and 36 months of age, catabolic stress such as febrile illness can trigger acute encephalopathic crisis (AEC) with bilateral striatal stroke‐like injury. AEC is the main prognostic determinant in GA1, often resulting in irreversible, dystonia‐predominant movement disorder (MD). Beside AECs, more attenuated “insidious‐onset” cases have been described, wherein patients commonly develop striatal lesions restricted to the dorsolateral putamen and delayed, less severe MD [9, 10]. While most patients are diagnosed by NBS or develop symptoms during childhood, some HE individuals are identified later through targeted metabolic diagnostics (TMD) in adolescence and adulthood. These “late‐diagnosed” patients often present with nonspecific neurologic symptoms such as headache, dementia, or coordination deficits. Cerebral MRI shows frontotemporal hypoplasia characteristic of GA1 as well as extrastriatal (but no striatal) abnormalities, including white matter changes and subependymal nodules [11, 12]. Additionally, three cases of malignant brain tumors have been reported in individuals not treated according to recommendations [13]. More recently, the clinical phenotype of GA1 has broadened to include chronic kidney disease, progressive white matter changes, and cognitive dysfunction in the HE group [14, 15, 16].

Metabolic treatment has proven effective over the past three decades, prompting evidence‐based recommendations and multiple updates [17, 18, 19, 20]. Metabolic maintenance treatment (MT) includes a low‐lysine diet in combination with a lysine‐free, tryptophan‐reduced, arginine‐fortified amino acid supplementation, and lifelong carnitine. Emergency treatment is advised during catabolic episodes. Tandem mass spectrometry‐based NBS, introduced in the 1990s, allows presymptomatic diagnosis and early treatment. Numerous prospective studies and a meta‐analysis have confirmed its major impact on preventing striatal injury and improving neurologic outcomes [5, 21].

In contrast to striatal and extrastriatal CNS manifestations, involvement of the peripheral nervous system (PNS) has not been systematically studied in GA1. To date, only a single case report has described peripheral neuropathy in an untreated, high‐excreting adult with late‐diagnosed GA1 [22]. MR neurography (MRN) is a novel, non‐invasive imaging technique that enables high‐resolution visualization of peripheral nerves and detection of structural lesions, even at the fascicular level [23, 24]. When combined with advanced methods such as magnetization transfer imaging (MTI) and diffusion tensor imaging (DTI), MRN allows sensitive assessment of microstructural nerve integrity [25]. MTI quantifies the magnetization transfer ratio (MTR), which reflects the interaction between macromolecule‐bound and free water protons and serves as an indirect marker of myelin integrity [26]. This technique has proven sensitive in detecting subclinical neuropathy, including presymptomatic nerve involvement in both hereditary and acquired neuropathies [27, 28].

In this study, we therefore systematically evaluated PNS morphology and function in a cohort of early‐treated GA1 patients identified by NBS and later‐diagnosed patients identified by targeted metabolic diagnostics using a multimodal approach based on clinical examination, high‐resolution MR neurography (MRN), brain MRI, and neurophysiological studies. Specifically, we analyzed (1) whether peripheral nerve alterations differ between LE and HE patients, (2) whether early diagnosis by NBS and treatment mitigates PNS damage in HE patients compared to those identified by targeted metabolic diagnostics, and (3) how peripheral nerve findings relate to other disease parameters, including clinical phenotype, biochemical metabolites, and extrastriatal abnormalities.

## 2 Materials and Methods

### 2.1 Study Population

This prospective, cross‐sectional, non‐randomized, controlled observational study included affected individuals if (1) GA1 was identified by NBS or TMD (i.e., before/after onset of neurologic symptoms), (2) diagnosis was confirmed by quantitative analysis of organic acids in urine and/or blood, genetic analysis of *GCDH* gene, and/or GCDH enzyme analysis in leukocytes or fibroblasts, (3) cerebral MRI and MRN could be performed without sedation (age > 6 years), and (4) written consent from participants, patients and/or parents was available. All patients were included in the European registry and network for Intoxication type Metabolic Diseases (E‐IMD; https://www.eimd‐registry.org) [29]. A control group consisted of 21 healthy, age‐matched individuals.

### 2.2 Clinical Assessments

Clinical manifestations, including neuropathic signs, were assessed during regular follow‐up investigations. Motor function was classified as impaired in patients with MD and as normal in those without. Onset of MD was defined either as (1) AEC or (2) insidious without apparent crisis. Assessment of peripheral neuropathy included (1) a targeted history focusing on muscle weakness, paresthesia, burning sensations, and pain as well as (2) a comprehensive neurological examination evaluating fine motor skills and coordination, muscle strength, muscle tone, deep tendon reflexes, and sensory function (pain, temperature, tactile [sharp/blunt] discrimination, and proprioception).

### 2.3 Biochemical Subtype

The biochemical phenotype was classified as HE or LE according to established criteria [8]. In LE patients, GA excretion was consistently < 100 mmol/mol creatinine, whereas HE patients had peak urinary GA concentrations > 100 mmol/mol.

### 2.4 Treatment and Metabolic Control

MT according to guideline recommendations consisted of (1) an age‐adapted low‐lysine diet with supplementation of a lysine‐free, tryptophan‐reduced, and arginine‐fortified amino acid supplement until the age of 6 years; (2) a protein‐controlled diet avoiding products with high lysine content and excessive protein intake for patients over the age of 6 years; and (3) oral carnitine supplementation. For NBS patients, adherence to MT until the age of 6 years was evaluated and defined as adequate if recommendations were followed continuously.

### 2.5 Magnetic Resonance Neurography (MRN)

Peripheral nerve imaging was conducted using a 3.0T MRI‐scanner (Magnetom Prisma^fit^, Siemens Healthineers) and a 15‐channel transmit‐receive knee coil (Siemens Healthineers) positioned at the right distal thigh. This anatomical location was chosen due to its known susceptibility to nerve lesions in various polyneuropathies [30, 31]. The MRN protocol included (1) an axial fat‐suppressed T2‐weighted 2D turbo spin‐echo (TSE) sequence, (2) two axial 3D proton density‐weighted gradient‐echo (GRE) FLASH sequences (with/without off‐resonance saturation pulse: Gaussian envelope, duration 9984 μs, frequency offset 1200 Hz) for magnetization transfer imaging (MTI), and (3) a single‐shot echo planar imaging (EPI) diffusion‐weighted sequence for diffusion tensor imaging (DTI). Sequence details are provided in Table S1.

Images were pseudonymized and analyzed by an experienced neuroradiologist (F.P.), using manual region‐of‐interest (ROI) tools in OsirixMD (Pixmeo Sarl, Switzerland). The tibial portion of the sciatic nerve was delineated on the high‐resolution T2‐weighted images, and ROIs were then transferred to the GRE‐images for MTI analysis, similar to previous investigations [32]. Any potential misalignment of ROI positions between the two sequences, such as that caused by patient movement, was manually corrected, if necessary. Subsequently, the magnetization transfer ratio (MTR) was calculated as: $\mathrm{MTR}=100\times \frac{S_{0}-S_{1}}{S_{0}}$, where $S_{0}$ and $S_{1}$ are mean signal intensities without ($S_{0}$) and with ($S_{1}$) the saturation pulse, respectively. MTR values were available for all but one patient, due to technical failure of fat saturation during acquisition. For DTI, ROIs of the tibial portion of the sciatic nerve were copied onto the corresponding b0‐image, and fractional anisotropy (FA) maps were generated using the DTI map plugin with a noise threshold of 10 (a.u.), as previously described for MRN [32, 33]. FA values were evaluated only within the patient cohort and not compared to controls. To limit B1‐field‐related bias, only the 10 (MTI) and 20 (DTI) central slices of each image slab were analyzed. Additionally, T2‐weighted images were qualitatively assessed for nerve enlargement or fascicular hyperintensities.

### 2.6 Cerebral MRI

As part of the study, each patient underwent a routine brain MRI (including T1w‐, T2w‐, and FLAIR sequences) that was evaluated by two experienced neuroradiologists (IH, FP) for pathological findings, in particular acute or chronic striatal lesions, white matter signal abnormalities, frontotemporal hypoplasia, and subependymal nodules. Findings were recorded on a per‐patient basis for correlation with individual MRN data.

### 2.7 Neurophysiological Studies

Neurophysiological examinations were performed in 17/21 patients (81%) on both lower extremities and included: motor nerve conduction with (1) compound muscle action potential (CMAP) amplitudes (mV) and (2) motor nerve conduction velocities (NCV) (m/s) of the tibial and peroneal nerves; sensory nerve conduction with (3) sensory nerve action potential (SNAP) amplitude (μV) and sensory NCV (m/s) of the sural nerve and somatosensory evoked potentials (SEPs) with (4) tibial nerve SEPs (latency, ms) recorded at the popliteal fossa (N8), lumbar spine (N22), and cortex (P40). Neurophysiological studies were performed in study patients only, while healthy controls did not undergo electrophysiological testing. All results were interpreted by a pediatric neurologist specialized in metabolic neuropathies. Each analysis was classified as normal (within ±2 SD), borderline (> 2–3 SD), or abnormal (> 3 SD) by comparison to standardized age‐matched reference ranges [34].

### 2.8 Statistical Analysis

Analyses were performed using R (version 4.5.0). Descriptive statistics were used to summarize continuous variables. Group comparisons (LE vs. HE, and NBS vs. TMD patients) were performed using the Mann–Whitney *U* test, given the small sample size. Associations between MRN metrics and clinical or biochemical variables were assessed by Spearman rank correlation.

To explore the combined discriminatory value of imaging, neurophysiology, and clinical variables, we conducted linear discriminant analyses (LDA), reporting accuracy and F1 scores. Across multivariate and discriminant models, the following variables were included depending on the model specification: sciatic nerve MTR and FA values, all neurophysiologic parameters (CMAP amplitudes, NCV, SNAP amplitudes, SEP latencies), urinary GA and 3‐OH‐GA concentrations, age at MRI/MRN, biochemical subtype, mode of diagnosis (NBS vs. TMD), and presence of neuropathic symptoms. The LDA was performed using the lda() function from the R package “MASS” (Version 7.3‐65).

Additional analyses included Poisson regression to model the association between subependymal nodules and sciatic nerve MTR, and binary classification tree analysis (R function ctree(), *partykit* package, version1.2‐24 [35]) to estimate an MTR cut‐off discriminating patients with and without nodules. Given the exploratory nature of the study, all *p* values should be regarded as descriptive measures.

## 3 Results

### 3.1 Patient Characteristics

In total, 21 patients (9 male, 12 female) from three metabolic centers were enrolled. Median age was 21 years in GA1 patients (range 7–45, interquartile range [IQR] 17.5–30) and 23 years in the control group (range 7–41, IQR 16.5–30.5). A total of 11 patients (52%) were identified by NBS and 10 (48%) by TMD. Clinical and biochemical characteristics are summarized in Table 1. The phenotype was HE in 15 patients (71%), and LE in six patients (29%). LE patients (mostly NBS) had a lower median age (14.0 years, IQR 10–18) than HE patients (25.0 years, IQR 19–23, *p* = 0.02). The NBS subgroup contained both HE (*n* = 5) and LE (*n* = 6) patients, whereas the TMD subgroup (*n* = 10) consisted primarily of HE patients (9 HE, 1 LE).

**TABLE 1:** Clinical, biochemical and neuroimaging characteristics.

Patient	Sex	Bio‐chemical subtype	Diagnosis mode	Age group at diagnosis	Age group at study (years)	GA (mmol/mol creatinine)	3‐OH‐GA (mmol/mol creatinine)	Clinical signs of PNP	Movement disorder	WM changes	GM changes	Fronto‐temporal hypoplasia	Subependymal lesions (*n*)	MTR sciatic nerve (%)	FA sciatic nerve
1	M	LE	NBS	Newborn	10–19	3.6	14.9	n	n	n	n	n	n	29.90	0.631
2	F	LE	NBS	Newborn	0–9	0.6	12.3	n	n	n	n	n	n	29.65	0.701
3	F	LE	NBS	Infancy	10–19	6.7	44.3	n	n	n	n	y	n	28.92	0.659
4	M	LE	NBS	Newborn	20–29	2.2	42.1	n	n	n	n	n	n	29.34	0.580
5	M	HE	TMD	Childhood	30–39	1042.2	46.3	n	y	y	y	y	y (2)	23.41	0.495
6	F	HE	NBS	Newborn	10–19	731	83.2	n	n	y	n	y	n	27.20	0.521
7	F	HE	NBS	Newborn	0–9	1522	121.5	n	n	y	n	y	y (1)	27.45	0.664
8	M	LE	TMD	Infancy	20–29	4	62.8	n	n	n	n	n	n	n.a.	0.497
9	F	LE	NBS	Newborn	10–19	2	35.9	n	y	n	y	n	n	30.31	0.528
10	F	HE	TMD	Adulthood	20–29	993.5	127.9	y (s)	n	y	n	y	y (2)	23.57	0.543
11	F	HE	TMD	Adulthood	40–49	209.9	142.2	n	n	n	n	y	y (10)	25.19	0.447
12	F	HE	TMD	Adulthood	40–49	1689.1	84.8	y (sm)	n	y	y	y	n	24.63	0.491
13	M	HE	NBS	Newborn	10–19	1466	98	n	n	y	y	y	y (2)	25.26	0.435
14	M	HE	TMD	Adolescence	30–39	2326.3	76.8	y (m)	n	y	n	y	y (26)	23.05	0.458
15	M	HE	TMD	Infancy	30–39	949.4	78.8	n	n	y	n	y	y (8)	23.57	0.380
16	M	HE	TMD	Childhood	20–29	1056.9	141.9	n	y	y	y	y	y (12)	24.61	0.407
17	M	HE	TMD	Newborn	20–29	2817.1	113.3	n	y	y	y	y	y (1)	28.95	0.595
18	F	HE	NBS	Infancy	10–19	43.5	58.4	n	n	y	n	y	y (5)	26.32	0.434
19	F	HE	TMD	Infancy	20–29	1395.2	117.6	n	n	y	y	y	y (5)	29.47	0.570
20	F	HE	TMD	Childhood	20–29	1700.9	81.6	n	n	y	y	y	y (10)	26.89	0.542
21	F	HE	NBS	Newborn	10–19	1917.3	130.9	y (m)	n	y	n	y	y (1)	25.77	0.500

*Note:* Age group at diagnosis is grouped as Newborn (≤ 1 month), Infancy (2–36 months), Childhood (3–10 years), Adolescence (11–17 years) and Adulthood (≥ 18 years).

Abbreviations: 3‐OH‐GA, 3‐hydroxyglutaric acid; FA, fractional anisotropy; GA, glutaric acid; GM, grey matter; HE, high excretor; LE, low excretor; MTR, magnetization transfer ratio; n.a., not applicable; PNP, polyneuropathy (classified as sensory [s], motor [m], or sensorimotor [sm]); WM, white matter.

In GA1 patients, median age of diagnosis was 2.8 months (range 0.03–420, IQR 0.23–78). Patients in the NBS group were younger at evaluation than the TMD group (median 18 vs. 30 years; *p* < 0.001). Median age at diagnosis in the NBS group was 0.23 months (range 0.03–2.8, IQR 0.17–0.37), vs. 6.5 years (range 0.25–35, IQR 2.4–28.3) in the TMD group (*p* < 0.0001).

In the NBS subgroup, 9/11 (82%) patients adhered to guideline‐recommended MT until age six, while two (18%) deviated, mainly from dietary guidance. After the age of 6 years, all but one (10/11) NBS patient followed a protein‐controlled diet as recommended. Among TMD patients, 6/10 followed dietary recommendations after diagnosis, whereas 4/10 reported no adherence.

No patient in either group experienced AEC. Four of 21 patients (19%) developed insidious‐onset MD, whereas 17 (81%) had no motor signs or symptoms. At diagnosis, all patients identified by NBS were asymptomatic. At last follow‐up, 9/11 NBS patients remained without MD, while two had developed mild to moderate MD of insidious‐onset. In the TMD group, 2/10 patients exhibited insidious‐onset MD, while the remaining eight had no motor symptoms.

### 3.2 GA1 Patients vs. Healthy Controls

On MRN, GA1 patients exhibited lower MTR values in the sciatic nerve compared to controls (26.6% [95% confidence interval (CI) 24.6–29] vs. 28.9% [95% CI 28.5–29.6]; *p* = 0.003), indicating microstructural nerve alterations. Neurophysiologic data were analyzed only within the patient cohort; therefore, no electrophysiological comparison with controls was performed.

### 3.3 Impact of Biochemical Subtype on MRN and Neurophysiology

**FIGURE 1:**
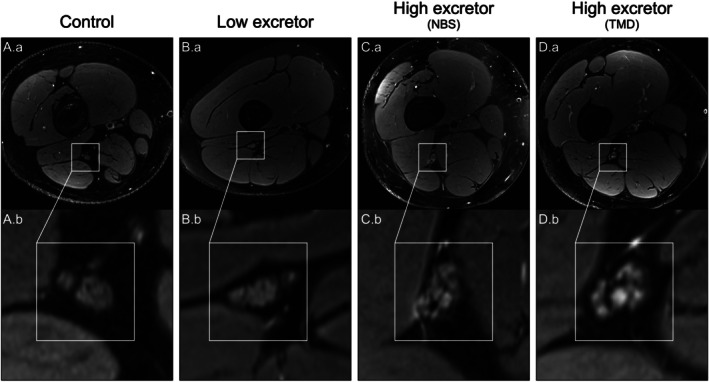
Morphological alterations of the sciatic nerve in GA1 patients compared to healthy controls. Axial fat‐saturated T2‐weighted MR neurography images of the right distal thigh in a healthy control (A), a low excretor (LE) GA1 patient (B), a high excretor (HE) patient identified by newborn screening (NBS) (C), and a HE patient identified via targeted metabolic diagnostics (TMD) (D). The upper panels (A.a–D.a.) display representative axial slices at the distal thigh level, whereas the lower panels (A.b–D.b.) present corresponding magnified insets highlighting the tibial and peroneal portions of the sciatic nerve. While nerve morphology appears normal in the control and LE patient, both HE patients (C, D) exhibit signs of polyneuropathic changes, including fascicular T2 hyperintensities and mild fascicular nerve enlargement, with the most pronounced alterations seen in the TMD‐diagnosed HE patient.

MRN revealed structural peripheral nerve differences between HE and LE patients. On qualitative assessment, 9/15 HE patients (60%, 6 TMD, 2 NBS) demonstrated mild diffuse enlargement and fascicular T2 hyperintensities of the sciatic nerve, consistent with polyneuropathy, whereas none of the six LE patients showed abnormal nerve appearance on MRN (Figure 1).

**FIGURE 2:**
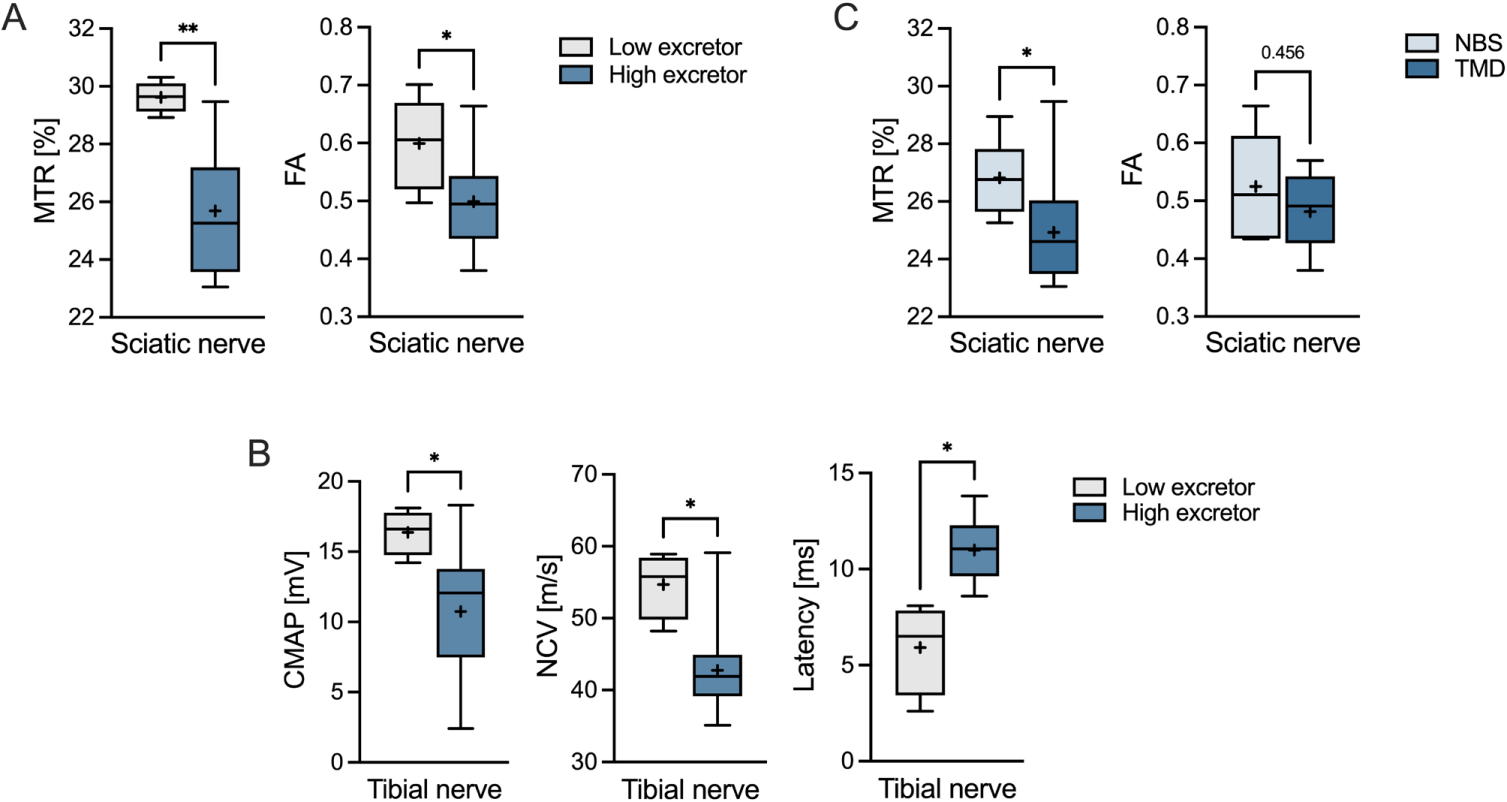
Descriptive statistics for MRN and neurophysiology findings across GA1 subgroups. (A) Magnetization transfer imaging and diffusion tensor imaging parameters (MTR, magnetization transfer ratio; FA, fractional anisotropy) in the sciatic nerve comparing low excretor (LE) and high excretor (HE) patients. (B) Neurophysiologic parameters (CMAP, compound muscle action potential; NCV, nerve conduction velocity; somatosensory evoked potential N8 latency) in the tibial nerve by biochemical subtype. (C) MTR and FA parameters within the HE subgroup, comparing patients identified by newborn screening (NBS) versus targeted metabolic diagnostics (TMD). Boxplots indicate median (line), mean (plus), and interquartile range (*p* < 0.05 [*], *p* < 0.01 [**]).

Additionally, subgroup comparison for quantitative measurements revealed lower MTR values in HE vs. LE patients for the sciatic nerve (HE: 25.3% [95% CI 23.6–27.2] vs. LE: 29.7% [95% CI 28.9–30.3]; *p* = 0.003). Similarly, median FA values of the sciatic nerve were lower in HE (0.5 [95% CI 0.44–0.54]) compared to LE patients (0.63 [95% CI 0.53–0.7]; *p* = 0.01) (Figure 2A). Based on these MRN findings, LDA analysis accurately classified biochemical phenotypes (accuracy = 1.0; F1 = 1.0).

Multivariate analysis revealed a positive correlation between urinary GA concentrations and MTR values (*p* = 0.026), whereas no concentration‐dependent association could be demonstrated for urinary 3‐OH‐GA levels (*p* = 0.312).

Overall, neurophysiologic studies were normal in all LE patients (3/3, 100%), whereas the majority of HE patients (11/14, 79%) showed borderline or abnormal findings in one or more parameters. Specifically, five HE patients (3 NBS, 2 TMD) revealed borderline findings, while six HE patients (all TMD) exhibited abnormal values in at least one neurophysiologic measure. Individual parameters were unavailable in a subset of cases due to technical limitations or incomplete cooperation during the examination. Complete neurophysiology results for all patients alongside reference ranges and missing values are listed in Table S2.

Compared to LE patients, HE patients revealed reduced median CMAP amplitudes (HE: 12.1 mV [95% CI 6.8–14] vs. LE: 16.8 mV [95% CI 14.2–18.1]; *p* = 0.038) and decreased median motor NCV of the tibial nerve (HE: 41.9 m/s [95% CI 36.9–45.6] vs. LE: 56.9 m/s [95% CI 48.2–58.9]; *p* = 0.021) (Figure 2B). Likewise, HE patients showed decreased median motor NCV of the peroneal nerve (HE: 43.4 m/s [95% CI 39.2–49.7] vs. LE: 53.7 m/s [95% CI 46.9–57.7]; *p* = 0.047). Median SEP latency reaction times of the tibial nerve were slower in HE patients (N8 popliteal region: HE: 11.1 ms [95% CI 9.6–12.5] vs. LE: 7.1 ms [95% CI 2.6–8.1]; *p* = 0.011) (Figure 2B). Similar results were obtained for N22 (lumbar vertebrae 1) and P40 (cortical) SEP markers. Additionally, median SNAP amplitudes in the sural nerve were decreased in the HE group, compared to LE (HE: 10.2 μV [95% CI 5.1–17.6] vs. LE: 25.6 μV [95% CI 20.4–33.2]; *p* = 0.011), while median sensory NCV showed a trend for slower velocities in the HE group (*p* = 0.085). In line with this, LDA analysis reliably predicted classification of biochemical phenotype by 100% (accuracy = 1.0; F1 = 1.0).

### 3.4 Correlation of MRN With Neurophysiological Parameters

Overall, MRN and neurophysiological findings were closely correlated. Specifically, sciatic nerve MTR was positively correlated with tibial CMAP (*r* = 0.87, *p* < 0.001), sural SNAP (*r* = 0.85, *p* < 0.001), and sensory NCV (*r* = 0.55, *p* = 0.037), and negatively correlated with SEP latency (N8 popliteal: *r* = −0.75, *p* = 0.002). Sciatic nerve FA was similarly correlated with tibial CMAP (*r* = 0.67, *p* = 0.004) and sural SNAP (*r* = 0.79, *p* = 0.001), and negatively with tibial SEP latency (N8 popliteal: *r* = −0.77, *p* = 0.001).

### 3.5 Correlation of Neuropathic Symptoms With MRN and Neurophysiology

Neuropathic symptoms were present in four patients (19%, 3 TMD, 1 NBS), all from the HE group and without a history of MD. Symptom patterns were as follows: Patient #10 reported distal paresthesia and numbness in the hands and feet, in line with a sensory neuropathy. Patient #12 described proximal leg weakness accompanied by diffuse numbness and bilateral intermittent burning pain in the thighs, compatible with a sensorimotor neuropathy. Patients #14 and #21 exhibited mild intention tremor and subtle fine‐motor coordination deficits without sensory complaints, consistent with a mild motor neuropathy. In all four individuals, muscle tone and deep tendon reflexes were normal, and none showed dystonic posturing or other central movement disorder features. The remaining 17 patients (81%) were clinically asymptomatic.

There was no significant age difference between symptomatic and asymptomatic individuals. However, patients with symptomatic neuropathy exhibited lower median MTR values in the sciatic nerve (24.1% [95% CI 23.1–25.8]) compared to asymptomatic individuals (27.3% [95% CI 25.2–29.5]; *p* = 0.038). For clinical context, healthy controls showed a higher median MTR of 28.9% [95% CI 28.5–29.6], placing symptomatic patients at the lowest end of the observed MTR range. FA values did not differ between groups (*p* = 0.55). Notably, the presence of neuropathic symptoms was not associated with differences in neurophysiological parameters. LDA analysis using symptom presence as a predictor yielded lower classification performance compared to models based on MRN or neurophysiology (accuracy = 0.8; F1 = 0.8).

### 3.6 Impact of Age on MRN and Neurophysiology

Multivariate ANOVA revealed negative effects of age on sciatic nerve MTR (*p* = 0.001) and FA values (*p* = 0.002). Notably, MTR values lower than 25.2% were exclusively observed in GA1 patients over 25 years of age (*p* = 0.007), whereas the lowest MTR value recorded in age‐matched healthy controls was 27.1%. However, LDA exclusively using age at MRI as a predictor revealed inferior classification performance (accuracy = 0.8; F1 = 0.86) compared to models incorporating MRN and neurophysiological data, suggesting that age alone does not sufficiently account for the observed differences in MRN parameters between biochemical subgroups.

### 3.7 Impact of Mode of Diagnosis on MRN and Neurophysiology

HE patients identified by NBS (6/15, 40%) exhibited higher median sciatic nerve MTR values compared to those identified through TMD (9/15, 60%) (NBS: 26.8% [95% CI 25.3–29] vs. TMD: 24.6% [95% CI 23.4–26.9]; *p* = 0.039). No significant differences in FA were found between groups (*p* = 0.46) (Figure 2C). LDA analysis based on MRN data classified the NBS and TMD subgroups with 80% accuracy (F1 = 0.73).

Compared to MRN findings, neurophysiological differences between NBS and TMD HE patients were less pronounced with a trend toward higher peroneal nerve CMAP amplitudes in the NBS group (6.8 mV [95% CI 4.7–8.3]) compared to TMD patients (3.2 mV [95% CI 2.4–6.1]; *p* = 0.066). No significant differences were observed in the remaining neurophysiological parameters.

### 3.8 Impact of Extrastriatal MRI Abnormalities

CNS abnormalities on MRI were frequent in HE patients and almost absent in LE patients. Specifically, white matter T2 hyperintensities (14/15, 93%) and subependymal periventricular nodules (13/15, 87%) were commonly noted in HE patients but absent in LE patients. Frontotemporal hypoplasia was observed in all 15 HE patients and in only one LE patient (17%). Acute striatal lesions were not observed. However, all four HE patients in the TMD group showed chronic scars in the dorsolateral putamen, consistent with prior insidious injury. MRN and brain MRI findings are summarized in Table 1.

**FIGURE 3:**
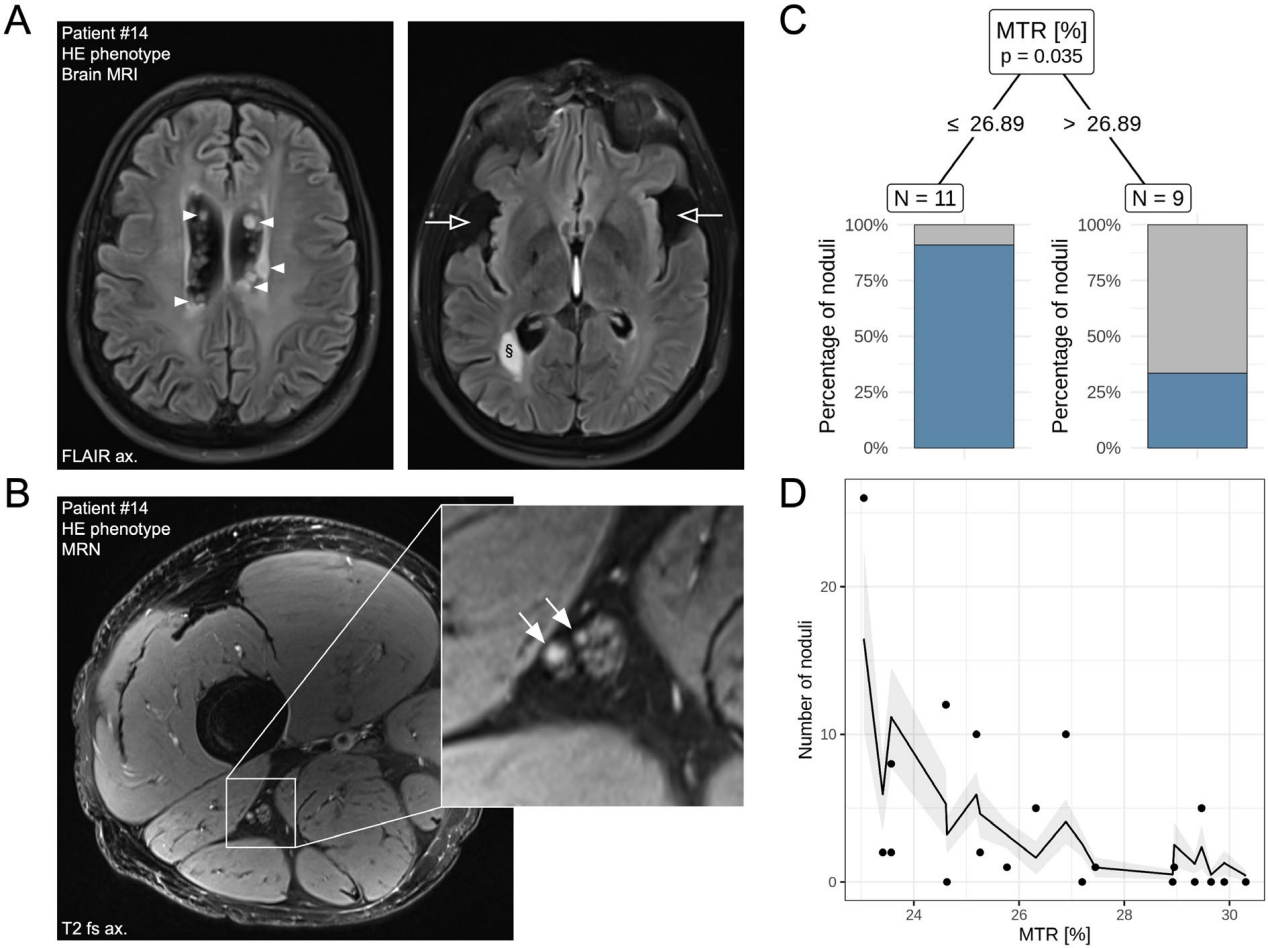
Association between subependymal nodules and peripheral neuropathy in HE patients. (A) Axial FLAIR images of a representative high excretor (HE) patient reveal multiple subependymal nodules along the lateral ventricles (arrowheads), asymmetric focal white matter hyperintensities (§) and fronto‐temporal hypoplasia (open arrow pointing at widened Sylvian fissures). (B) Corresponding axial T2‐weighted MR neurography of the same patient reveals fascicular T2 hyperintensities in the sciatic nerve (solid arrows), consistent with polyneuropathic changes. (C) Binary classification tree based on sciatic nerve MTR values identifies a cut‐off at 26.89%, separating patients with and without subependymal nodules. Nodules were present in 10/11 patients with MTR ≤ 26.89%, compared to 2/9 patients with MTR > 26.89% (*p* = 0.035). (D) Poisson regression analysis demonstrates an inverse relationship between sciatic nerve MTR and the number of subependymal nodules. The black line depicts the fitted model with 95% confidence intervals shown in grey.

In general, we observed notable associations between extrastriatal MRI abnormalities and PNS alterations. Patients with frontotemporal hypoplasia demonstrated lower sciatic nerve MTR (25.5% [95% CI 23.6–27.5]) and FA (0.5 [95% CI 0.44–0.57]) values compared to patients without (MTR: 29.8% [95% CI 29.3–30.3]; *p* = 0.004, FA: 0.61 [95% CI 0.53–0.7]; *p* < 0.05), respectively. Similar results were found for sciatic nerve MTR in patients with (25.5% [95% CI 23.6–27.5]) and without (29.5% [95% CI 25.2–30.3]; *p* = 0.012) T2‐hyperintense white matter changes. These findings were not age‐related. Subependymal nodules were present in 13/21 patients (62%) and were more frequent in patients with sciatic nerve MTR values below 26.9% (10/11, 91%; *p* = 0.035) (Figure 3A–C). Poisson regression revealed a negative association of nodule count and sciatic nerve MTR values (Figure 3D).

### 3.9 Impact of Striatal Injury and Motor Phenotype

Striatal or other gray matter abnormalities were not related to MRN or neurophysiology findings. Likewise, MD (present in 4/21 patients, 19%) had no impact on either of these examinations and did not correlate with extrastriatal abnormalities.

## 4 Discussion

This first systematic investigation of PNS involvement in GA1 yielded several key findings: (1) GA1 patients frequently exhibit PNS involvement; (2) the HE biochemical subtype was identified as the primary risk factor for clinical, structural, and neurophysiological PNS involvement, whereas LE patients remained unaffected; (3) MRN and neurophysiology predicted biochemical phenotype with 100% accuracy; (4) extrastriatal abnormalities and subependymal nodules, but not striatal injury or MD, were associated with MRN changes; (5) overt clinical PNS symptoms were rare but associated with abnormal MRN; and (6) early identification of GA1 by NBS resulted in milder MRN alterations in HE patients, while neurophysiological findings were unaffected.

Over the past four decades, the clinical spectrum of GA1 has progressively expanded. Initially, the description of the clinical phenotype was restricted to AEC, which remains the primary prognostic determinant [9, 36, 37]. Following implementation of NBS, early MT, and prospective long‐term studies, the occurrence of insidious‐onset striatal injury without AEC was increasingly recognized, often in the context of MT deviations [10, 15, 38]. More recently, extrastriatal CNS findings such as white matter abnormalities, subependymal nodules [11, 39], and enlargement of optic chiasm [40] have been described, though their clinical significance remains uncertain. However, HE patients more frequently and more extensively exhibit white matter changes and show lower IQ scores compared to LE individuals, suggesting a possible link between white matter abnormalities and cognitive function [16]. Hearing dysfunction after AEC has been reported, suggesting further neuronal damage [41]. Additionally, renal complications have emerged as the first extra‐neurological manifestation, even in individuals identified by NBS and prospectively treated [14].

To date, only a single case report of adult‐onset neuropathy and one on myeloneuropathy have linked GA1 to PNS involvement [22, 42]. In the current study, we not only document PNS involvement but also reveal clear differences between the biochemical subtypes: whereas the majority of HE patients (60%) showed abnormal PNS findings on MRN, all LE patients had normal examinations. Neurophysiology further supported this distinction, as borderline or abnormal values occurred exclusively in HE patients, while most parameters were still within normal reference ranges and should therefore be interpreted with caution.

Although the biochemical phenotype in GA1 is genotype‐associated, it does not predict the risk for striatal injury as both LE and HE patients are equally vulnerable if untreated [9, 21, 43]. This has been attributed to intracerebral *de novo* synthesis and entrapment of neurotoxic metabolites due to limited blood–brain barrier permeability for dicarboxylic acids like GA and 3‐OH‐GA [44], supported by postmortem cerebral metabolite levels [45]. However, emerging data suggest that HE and LE phenotypes differ in their disease course. HE patients show larger head circumference [46], lower cognitive performance [16], higher risk of early subdural effusions [47], more frequent white matter abnormalities [11, 12], and elevated intracerebral concentrations of neurotoxic metabolites [39]. Subependymal nodules, unique to HE and developing in the second decade, progress slowly with age and were also noted in the first reported GA1 patient with polyneuropathy [11, 22].

In line with these findings, we frequently observed extrastriatal abnormalities such as frontotemporal hypoplasia and white matter changes in HE patients in this study that correlated strongly with MRN abnormalities, suggesting a shared underlying mechanism involving progressive axonal and/or myelin damage in both CNS and PNS.

In contrast, striatal injury or MD shared no association with MRN findings. This dissociation of CNS and PNS abnormalities suggests that peripheral neuropathy and AEC follow distinct pathophysiological trajectories. While AEC results from acute, localized toxicity regardless of biochemical subtype [9, 21, 43], chronic extrastriatal manifestations—including PNS involvement—appear specific to HE and are likely driven by long‐term metabolite accumulation. Proposed mechanisms of chronic toxicity from GA and 3‐OH‐GA include citrate cycle inhibition [3], altered anaplerotic flux between astrocytes and neurons [48], neurotransmitter imbalance by activation of glutamatergic signaling via NMDA receptors [49], and oxidative stress [50]. More recently, selective glutarylation of mitochondrial proteins in astroglial cells impairing protein stability was described as an additional potential pathophysiological mechanism, further impairing glial support and neuronal energy homeostasis [51]. However, the exact link between these CNS‐focused mechanisms and PNS involvement remains unclear. We hypothesize that the observed MTR reductions in HE patients may partly reflect intramyelinic edema caused by Schwann cell impairment under chronic metabolic stress, leading to accumulation of osmotically active metabolites and subsequent water retention within the myelin sheath—analogous to postulated impaired siphoning of potassium and osmotically obliged water by the panglial syncytium in the CNS [52]. Intramyelinic edema may reduce macromolecular density and thus magnetization transfer, resulting in lower MTR values, even in the absence of overt demyelination [53]. The largely subclinical nature of peripheral neuropathy in GA1, despite clear imaging and neurophysiologic changes, may reflect slow, diffuse nerve involvement and the resilience of Schwann cells, with deficits remaining below the clinical threshold.

Our results suggest that early diagnosis via NBS may reduce the severity of PNS involvement, as HE patients identified by NBS showed better nerve integrity on MRN and milder neurophysiological alterations than those diagnosed later by targeted metabolic diagnostics. NBS has markedly improved neurologic outcomes in GA1 by reducing the incidence of striatal injury, morbidity, and mortality, as shown in multiple prospective studies [5, 9, 15, 38], and confirmed by a meta‐analysis [21]. Consequently, NBS is now strongly recommended [19]. However, current MT does not prevent extrastriatal or renal manifestations [15]. The more pronounced PNS and extrastriatal involvement in HE patients raises the question of whether tailored treatment strategies are needed for this subgroup. While prolonged lysine restriction beyond age six is sometimes pursued, albeit under a liberalized dietary regimen [19], no evidence currently supports intensified or subtype‐specific MT. Several abnormalities still emerge despite early and strict MT, highlighting its limited efficacy against non‐striatal manifestations. Thus, further research is required to evaluate the clinical relevance of chronic manifestations and to develop targeted treatment strategies.

This study has several limitations. First, the cohort size is small, which limits statistical power for subgroup analyses, including the assessment of dietary influences on long‐term PNS involvement, and the generalizability to all GA1 patients. Because only a few patients had documented non‐adherence to metabolic therapy, no meaningful evaluation of treatment quality was feasible. Thus, larger multi‐center collaborations and patient registries such as E‐IMD will be needed to gather larger cohorts. Second, the cross‐sectional design prevents any conclusion on the temporal evolution of PNS involvement; longitudinal studies are needed to determine whether subclinical abnormalities progress with age, particularly into later adulthood, as we cannot exclude the possibility that overt clinical neuropathy may emerge at older ages. Third, our cohort did not include patients with severe MD (e.g., severe AEC), as they were not able to undergo MRN without sedation. This may introduce some bias, as we rather selected a less severely affected subgroup of patients. In addition, age may be a confounding factor when assessing the impact of mode of diagnosis, as the NBS subgroup was younger compared to the subgroup identified by targeted metabolic diagnostics. However, age alone per se was not sufficient to explain the observed differences between HE and LE patients. Lastly, nerve biopsy, which could have provided valuable pathological correlation with MRN findings, was not performed due to its invasiveness and lack of clinical indication in this cohort.

## 5 Conclusion

In summary, our findings extend the phenotypic spectrum of GA1 by documenting frequent, predominantly subclinical peripheral nerve involvement in HE patients, most likely linked to chronic exposure to GA and related metabolites and independent of striatal injury. NBS and early treatment may reduce the severity of neuropathy, yet do not entirely avert it. These results should encourage clinicians to remain vigilant for PNS‐related symptoms in GA1 and underline the importance of new treatment strategies aiming at preventing chronic disease manifestations.

## Author Contributions

**Fabian Preisner:** study concept and design; drafting/revision of the manuscript for content; major role in the acquisition of data (MRN); analysis or interpretation of data. **Sven F. Garbade:** drafting/revision of the manuscript for content; analysis or interpretation of data. **Inga Harting:** drafting/revision of the manuscript for content; analysis or interpretation of data. **Saskia B. Wortmann:** drafting/revision of the manuscript for content; acquisition of data. **Chris Mühlhausen:** drafting/revision of the manuscript for content; acquisition of data. **Sabine Heiland:** drafting/revision of the manuscript for content; analysis or interpretation of data. **Martin Bendszus:** study concept and design; drafting/revision of the manuscript for content. **Stefan Kölker:** study concept and design; drafting/revision of the manuscript for content; analysis or interpretation of data. **Nikolas Boy:** study concept and design; drafting/revision of the manuscript for content; major role in the acquisition of data; analysis or interpretation of data. Fabian Preisner and Nikolas Boy are the guarantors for the article. All co‐authors approved the final version of the manuscript.

## Funding

Several participants are part of a prospective long‐term outcome study of patients identified by newborn screening in Germany, which is supported by the Dietmar Hopp Foundation, St. Leon‐Rot, Germany (NGS2020, grant no. 2311221, and NGS2025, grant no. 1DH2011117, to S.K.). The authors confirm independence from the sponsors. The content of the article has not been influenced by the sponsors.

## Ethics Statement

The Institutional Ethics Committee of the coordinating center and all contributing study sites approved the study (University Hospital Heidelberg, application nos. S‐525/2010, S‐398/2012, S‐020/2020).

## Consent

Written and informed consent was obtained from all participants. For participants under the age of 18, written and informed consent was obtained from their parents or legal guardians.

## Conflicts of Interest

The authors declare no conflicts of interest.

## Supporting information


**Table S1:** MR imaging parameters.
**Table S2:** Neurophysiological results.

## Data Availability

The data that support the findings of this study are available on request from the corresponding author. The data are not publicly available due to privacy or ethical restrictions.
