# Supplementary material for: Peripheral Neuropathy Expands the Neurological Phenotype in Glutaric Aciduria Type 1
Source: J Inherit Metab Dis. 2026 Jan 4;49(1):e70131. doi: 10.1002/jimd.70131 (PMC12765624; doi:10.1002/jimd.70131)
Supplement: Supplementary file 1 — Table S1: MR imaging parameters. Table S2: Neurophysiological results. [file JIMD-49-0-s001.docx]

**Supplementary Material**

Supplementary Table 1. MR imaging parameters

|  | **T2 weighted turbo spin echo (TSE)** | **Magnetization transfer imaging (MTI)** | **Diffusion tensor imaging (single-shot EPI)** |
| --- | --- | --- | --- |
| **Orientation** | Axial | Axial | Axial |
| **Repetition time (TR) [ms]** | 5970 | 50 | 5100 |
| **Echo time (TE) [ms]** | 54 | 4.92 | 74 |
| **Field of view [mm]** | 160 × 160 | 160 × 160 | 160 × 160 |
| **Matrix size** | 512 × 333 | 256 × 225 | 128 × 128 |
| **Slice thickness [mm]** | 4.0 | 4.0 | 4.0 |
| **Interslice gap [mm]** | 0.4 | n.a. | 0.4 |
| **Number of slices** | 24 | 16 | 24 |
| **Number of averages** | 3 | 1 | 3 |
| **Fat suppression** | Yes | Yes | Yes |
| **Echo train length** | 13 | n.a. | 45 |
| **Flip angle [°]** | 131 | 7 | 90 |
| **b-value 1 [s/mm^2^]** | n.a. | n.a. | 0 |
| **b-value 2 [s/mm^2^]** | n.a. | n.a. | 1000 |
| **Acquisition time** | 4 min 42 sec | 3 min 48 sec | 5 min 48 sec |

Magnetization transfer imaging consisted of two identical gradient-echo acquisitions with and without off-resonance saturation pulse. n.a., not applicable

Supplementary Table 2. Neurophysiological results

| **Patient** | **Sex** | **Bio-chemical subtype** | **Diagnosis mode** | **Age group at diagnosis** | **Age group at study**  [years] | **Clinical signs of PNP** | **Tibial CMAP amplitude** [mV] | **Peroneal CMAP amplitude** [mV] | **Tibial motor NCV** [m/s] | **Peroneal motor NCV** [m/s] | **Tibial SEP latency** [N8; ms] | **Sural SNAP amplitude** [µV] | **Sural sensory NCV** [m/s] |
| --- | --- | --- | --- | --- | --- | --- | --- | --- | --- | --- | --- | --- | --- |
| **1** | M | LE | NBS | Newborn | 10-19 | n | 16.8 (15.8±1.8) | 6.3 (7.2±1.6) | 58.9 (48.2±2.8) | 57.7 (49.6±3.4) | 7.1 (7.5±0.9) | 25.6 (18.7±4.4) | 48.7 (40.6±4.8) |
| **2** | F | LE | NBS | Newborn | 0-9 | n | 14.2 (15.8±1.8) | 4.9 (7.2±1.6) | 48.2 (48.2±2.8) | 46.9 (49.6±3.4) | 2.6 (6.2±0.6) | 33.2 (18.7±4.4) | 52.9 (40.6±4.8) |
| **3** | F | LE | NBS | Infancy | 10-19 | n | 18.1 (15.8±1.8) | 3.9 (7.2±1.6) | 56.9 (48.2±2.8) | 53.7 (49.6±3.4) | 8.1 (8.1±0.8) | 20.4 (18.7±4.4) | 46.6 (40.6±4.8) |
| **4** | M | LE | NBS | Newborn | 20-29 | n | n.t. | n.t. | n.t. | n.t. | n.t. | n.t. | n.t. |
| **5** | M | HE | TMD | Childhood | 30-39 | n | 6.8 (14.9±3.4) | 3.2 (8.7±2.1) | 44.3 (49.8±4.4) | 47.7 (48.8±3.5) | 11.3 (8.5±1) | 5.1 (21.2±5.3) | 43.3 (40.2±5.5) |
| **6** | F | HE | NBS | Newborn | 10-19 | n | 14.4 (14.9±3.4) | 6.6 (8.7±2.1) | 40.3 (49.8±4.4) | 47 (48.8±3.5) | 8.6 (8.5±1) | 15.3 (21.2±5.3) | 46.2 (40.2±5.5) |
| **7** | F | HE | NBS | Newborn | 0-9 | n | n.t. | n.t. | n.t. | n.t. | n.t. | n.t. | n.t. |
| **8** | M | LE | TMD | Infancy | 20-29 | n | n.t. | n.t. | n.t. | n.t. | n.t. | n.t. | n.t. |
| **9** | F | LE | NBS | Newborn | 10-19 | n | n.t. | n.t. | n.t. | n.t. | n.t. | n.t. | n.t. |
| **10** | F | HE | TMD | Adulthood | 20-29 | y | 7.7 (14.9±3.4) | 1.6 (8.7±2.1) | 36.8 (49.8±4.4) | 42 (48.8±3.5) | 9.6 (8.5±1) | 10.2 (21.2±5.3) | 35 (40.2±5.5) |
| **11** | F | HE | TMD | Adulthood | 40-49 | n | 8.7 (14.9±3.4) | 3.1 (8.7±2.1) | 36.9 (49.8±4.4) | 39.2 (48.8±3.5) | 11.1 (8.5±1) | 9.3 (21.2±5.3) | 41.3 (40.2±5.5) |
| **12** | F | HE | TMD | Adulthood | 40-49 | y | 12.1 (14.9±3.4) | 2.3 (8.7±2.1) | 40.7 (49.8±4.4) | 17.7 (48.8±3.5) | 13.8 (8.5±1) | n.a. | n.a. |
| **13** | M | HE | NBS | Newborn | 10-19 | n | 14 (14.9±3.4) | 6.9 (8.7±2.1) | 43.1 (49.8±4.4) | 44.2 (48.8±3.5) | n.a. | 17.6 (21.2±5.3) | 51.5 (40.2±5.5) |
| **14** | M | HE | TMD | Adolescence | 30-39 | y | 2.4 (14.9±3.4) | 6.1 (8.7±2.1) | 59.1 (49.8±4.4) | 30.8 (48.8±3.5) | 12.5 (8.5±1) | 4.7 (21.2±5.3) | n.a. |
| **15** | M | HE | TMD | Infancy | 30-39 | n | 10.3 (14.9±3.4) | 4.7 (8.7±2.1) | 39.9 (49.8±4.4) | 42.9 (48.8±3.5) | 13.3 (8.5±1) | 6.8 (21.2±5.3) | 39 (40.2±5.5) |
| **16** | M | HE | TMD | Childhood | 20-29 | n | 5.2 (14.9±3.4) | 2.9 (8.7±2.1) | 35.1 (49.8±4.4) | 49.9 (48.8±3.5) | 9.8 (8.5±1) | 4.7 (21.2±5.3) | 44.4 (40.2±5.5) |
| **17** | M | HE | TMD | Newborn | 20-29 | n | 18.3 (14.9±3.4) | 10.6 (8.7±2.1) | 40.4 (49.8±4.4) | 49.7 (48.8±3.5) | n.a. | 16.1 (21.2±5.3) | 52.9 (40.2±5.5) |
| **18** | F | HE | NBS | Infancy | 10-19 | n | 12 (14.9±3.4) | 4.9 (8.7±2.1) | 48 (49.8±4.4) | 51.6 (48.8±3.5) | 11 (8.5±1) | 5.3 (21.2±5.3) | 43.5 (40.2±5.5) |
| **19** | F | HE | TMD | Infancy | 20-29 | n | 13.7 (14.9±3.4) | 4.8 (8.7±2.1) | 43.7 (49.8±4.4) | 39.4 (48.8±3.5) | 9.7 (8.5±1) | 18.6 (21.2±5.3) | 35.5 (40.2±5.5) |
| **20** | F | HE | TMD | Childhood | 20-29 | n | 12.8 (14.9±3.4) | 3.2 (8.7±2.1) | 45.6 (49.8±4.4) | 43.9 (48.8±3.5) | 9.6 (8.5±1) | 20 (21.2±5.3) | 57.7 (40.2±5.5) |
| **21** | F | HE | NBS | Newborn | 10-19 | y | 12.1 (14.9±3.4) | 7.6 (8.7±2.1) | 44.7 (49.8±4.4) | 41.9 (48.8±3.5) | 11.6 (8.5±1) | 10.4 (21.2±5.3) | 44.3 (40.2±5.5) |

LE, low excretor; HE, high excretor; PNP, polyneuropathy; CMAP, compound muscle action potential; NCV, nerve conduction velocity; SEP, somatosensory evoked potential; SNAP, sensory nerve action potential; N8 recorded at the popliteal fossa (data for N22 recorded at the lumbar spine and P40 recorded at the cortex are not shown); n.t., not tested; n.a., data not available (not all neurophysiology measurements could be obtained in all patients due to technical limitations or incomplete patient cooperation). Age-matched normal reference ranges (in parentheses) are provided next to each individual value and were derived from Cai F, Zhang J. Study of nerve conduction and late responses in normal Chinese infants, children, and adults. *J Child Neurol.* 1997;12(1):13-8. Age group at diagnosis is grouped as Newborn (≤ 1 month), Infancy (2-36 months), Childhood (3-10 years), Adolescence (11-17 years) and Adulthood (≥ 18 years).
